# Acute Hemothorax Causing Hemorrhagic Shock Following Small-bore Thoracocentesis in a Patient on Clopidogrel: A Case Report and Literature Review

**DOI:** 10.7759/cureus.7431

**Published:** 2020-03-27

**Authors:** Rania Al Asmar, Fuad Zeid

**Affiliations:** 1 Internal Medicine, Marshall University, Joan C. Edwards School of Medicine, Huntington, USA; 2 Pulmonary Medicine, Marshall University, Joan C. Edwards School of Medicine, Huntington, USA

**Keywords:** thoracocentesis, hemothorax, hemorrhagic shock, clopidogrel, bleeding risk

## Abstract

Clopidogrel is one of the most commonly prescribed thienopyridines used postcoronary stenting for acute coronary syndrome (ACS). There have been several concerns regarding performing small-bore thoracocentesis on patients on clopidogrel in our practice. We present an 85-year-old male with a history of diabetes, atrial fibrillation, diastolic heart failure, chronic kidney disease (CKD) stage three, who recently had a non-ST elevation myocardial infarction (NSTEMI) requiring a drug-eluting stent (DES) to the left anterior descending (LAD) artery, and was on dual anti-platelet therapy (DAPT). He was admitted with worsening shortness of breath and found to have bilateral pleural effusions. He required several small-bore, ultrasound-guided thoracocentesis on the right side while on clopidogrel. Intensivists or residents did all the procedures. The patient went into a hemorrhagic shock following his third small-bore thoracocentesis, requiring pressors, blood and platelet transfusions, and a surgical intercostal (IC) drain insertion. He eventually became clinically stable. An IC arteriogram within 24 h ruled out IC artery injury. Healthcare professionals perceive small-bore thoracocentesis as a safe procedure done on medical, surgical, intensive care, and interventional radiology (IR) units. The overall consensus is that it is safe to perform it on patients taking clopidogrel. We emphasize through this case report the bleeding risks associated with performing such procedures on patients while on clopidogrel and considering holding clopidogrel when feasible or bridging with an intravenous anti-platelet drug.

## Introduction

It has been a controversial topic of whether clopidogrel is associated with significant bleeding risk in patients undergoing simple noninvasive procedures. Literature over the past 15 years was supportive of performing small-bore (<14 Fr), ultrasound-guided thoracocentesis while patients are on clopidogrel [1-5]. On the other hand, several studies published recently found a significant risk of bleeding from performing these procedures on such patients. Thus, they recommended holding antiplatelet therapy, when possible, five days before such procedures or conducting large randomized controlled trials (RCTs) to assess its safety [6-7]. The Society of Interventional Radiology (SIR) published guidelines in 2012 which stated that for Category 1 procedures, including thoracocentesis, clopidogrel should be held for one to five days prior to the procedure [8]. 

## Case presentation

**Figure 1:**
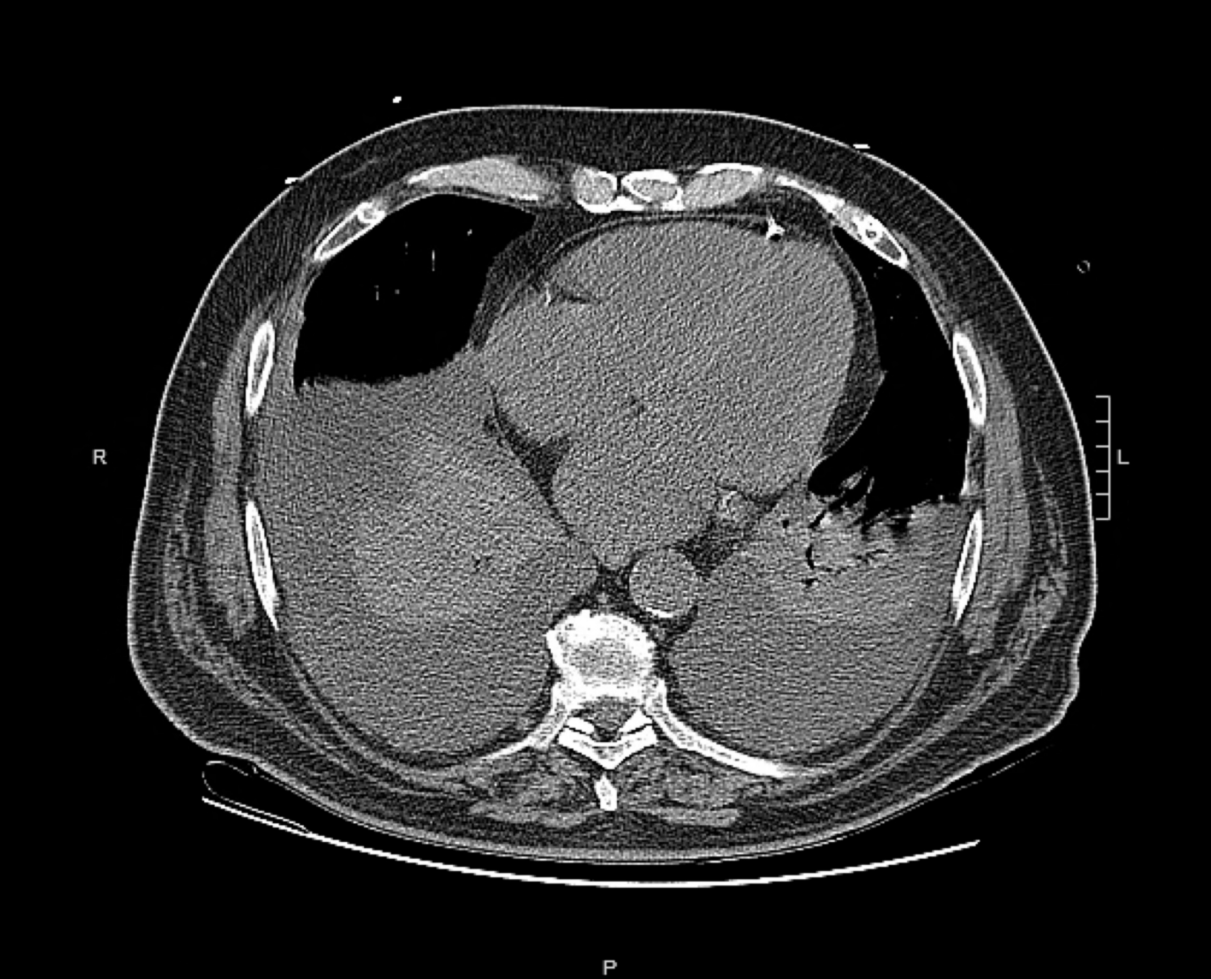
CT scan of the chest without contrast showing bilateral lung lobes atelectasis and significant pleural effusions.

**Figure 2:**
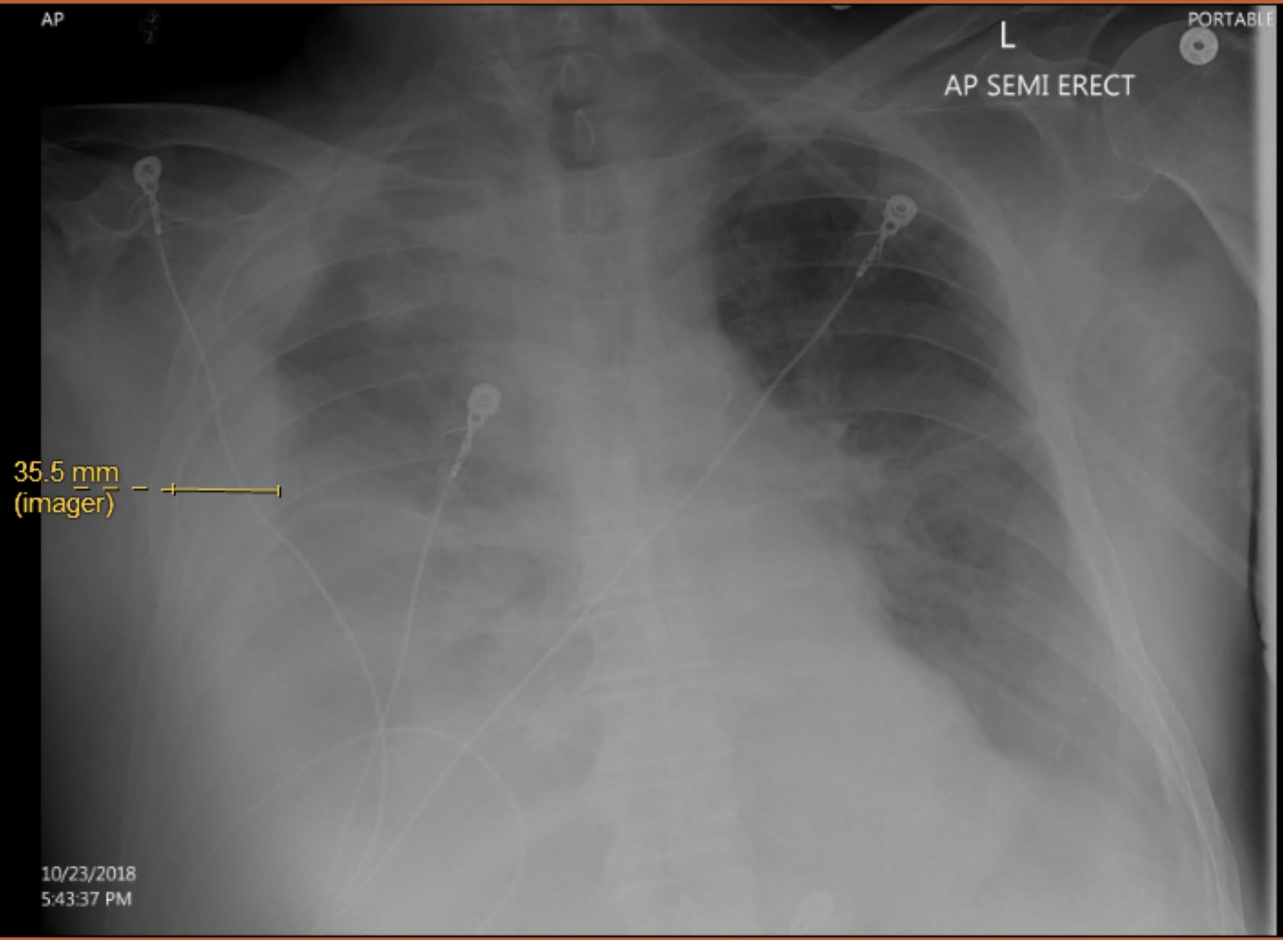
Repeat portable chest X-ray of the patient after clinical deterioration showing a new 3.5-cm right-sided hemothorax.

We present an 85-year-old male with a past medical history of obesity, type two diabetes, atrial fibrillation, diastolic heart failure, chronic kidney disease (CKD) stage three, and coronary artery disease. The patient had a history of non-ST elevation myocardial infarction (NSTEMI) four weeks before the current admission, requiring a drug-eluting stent (DES) to the left anterior descending (LAD) artery. He also had a history of significant gastrointestinal bleed in the past month, for which apixaban was stopped. The patient was admitted to our hospital with worsening shortness of breath and found to have bilateral pleural effusions, right greater than left. He was afebrile and did not have any symptoms of pneumonia. The patient was started on IV furosemide and had an initial diagnostic, small-bore, ultrasound-guided tap from the right pleural effusion that was uneventful and yielded straw-colored 1000 mL of fluid. The pleural fluid analysis was mildly exudative based on Light’s lactate dehydrogenase (LDH) criteria, but cytology was negative as well as Gram stain, bacterial, and fungal cultures. Autoimmune screening, including anti-nuclear antibody (ANA) and extractable nuclear antigen (ENA), was negative. In anticipation of a potential repeat pleural tap, the patient's aspirin was stopped. One week later, the patient was getting more short of breath, and chest X-ray revealed recurrent bilateral effusions worse on the right side. Echocardiogram showed features of diastolic dysfunction, with a left ventricular ejection fraction of 55% and no significant valvular disease. Arterial blood gas (ABG) was suggestive of hypercapnic respiratory failure; thus, he was started on non-invasive ventilation (NIV) and shifted to the ICU. He underwent a second, uneventful pleural tap on the right side, that yielded 1500 mL of straw-colored fluid. Analysis again showed an exudate with negative bacterial, fungal cultures, and cytology. CT scan of the chest showed basal atelectasis with significant pleural effusions, no lung masses, or lymph nodes enlargement (Figure 1). The patient was transferred to the step-down unit, completed a 10-day course of antibiotics for possible community-acquired pneumonia, although sputum and blood cultures remained negative. One week later, the patient again clinically deteriorated and was admitted to ICU with hypercapnic respiratory failure and worsening pleural effusions. He initially required continuous bilevel positive airway pressure (BiPAP) ventilation until he stabilized. He had a third, right-sided thoracocentesis under ultrasound-guidance from a posterior approach, atraumatic, and yielded 1500 mL of clear thin yellow fluid. The patient had a follow-up chest X-ray 20 min later that showed improvement in the previously seen right-sided pleural effusion and no pneumothorax. However, two hours later, the patient was suddenly getting sweaty, tachypneic, lethargic while on BiPAP. His blood pressure (BP) was reading 60/40 mmHg, heart rate was dropping to 40 bpm, and he was less responsive. There was minimal air entry on auscultating the right chest with dullness to percussion, and no signs of any pain or tenderness. ABG showed hypoxia and a stable PCO2 level. Immediate chest X-ray showed a new 3.5 cm hemothorax on the right side (Figure 2).

He was given 1 L of IV fluids, norepinephrine for BP support, and an urgent right-sided IC surgical drain was inserted, which immediately drained 1.7 L of blood.

The patient had a central line insertion, and three units of packed red blood cells (PRBC) transfused. Repeat hemoglobin after the second transfused unit was still below baseline at 6 g/L (from 8 g/L). He clinically started to improve after the hemothorax drainage, was more responsive, and pressor requirement was decreased. Overnight he received two units of platelets, and clopidogrel was stopped. The next day, the IC surgical drain still had around 800 mL output of serosanguinous fluid, but he was clinically back to his baseline as before this event. CT scan of the chest with IV contrast did not show any active bleeding into the pleural space but showed complex right-sided effusion. The IR team performed IC arteries angiogram to diagnose the site of the injured vessel. That was done within 24 h of the event and was negative. The cardiothoracic surgery team was consulted for possible video-assisted thoracoscopic surgery (VATS) procedure in the right pleural space. The patient continued to improve, and after a few days we removed the surgical drain, and transferred him out to the step-down unit with cardiothoracic surgical team following up on his care.

## Discussion

It is well established that thienopyridine anti-platelet agents, like clopidogrel and prasugrel, are very potent inhibitors of platelet activation and aggregation. They irreversibly inhibit P2Y12 receptors [9]. Thus, they imply a theoretical bleeding risk at all times. Their potency makes them the currently recommended anti-platelets for patients after myocardial infarction with or without coronary stent(s) placement. Several prospective cohort studies tried to conclude the safety of continuing clopidogrel in patients undergoing ultrasound-guided thoracocentesis. Still, they all came to different conclusions, with few of them reporting infrequent bleeding complications. Abouzgheib et al. have retrospectively looked into the safety of continuing clopidogrel in 24 patients getting ultrasound-guided chest tubes by studying bleeding complications. There were no bleeding events, and thus they concluded that clopidogrel remains a relative, but not an absolute, contraindication to chest tube insertion [2]. Also, Dammert et al. retrospectively reviewed their population of 30 patients who were on clopidogrel and underwent small-bore chest tube insertion by a pulmonologist. They found no significant bleeding events reported, even in the sickest 10 patients who were in ICU. Thus they concluded that the procedure has a lower risk of bleeding when done by experienced providers [3]. Zalt et al. came to the same conclusion after their prospective cohort study on 30 patients [5]. In addition, Mahmood et al. conducted a prospective cohort study assessing the risk of developing a hemothorax in patients on clopidogrel. Twenty-five patients on the latter drug who had thoracocentesis were compared to 50 patients who had thoracocentesis without being on clopidogrel. There was one reported case of hemothorax requiring two units of PRBC transfused with chest tube drainage in the clopidogrel group. The latter management strategy stabilized their patient, and the bleeding event was self-limited, just like in our presented patient. The authors agreed that larger RCTs are warranted to assess the safety of thoracocentesis while on clopidogrel [7]. Some hemothoraces after thoracocentesis result from a venous laceration. Physicians should keep in mind the possibility of anatomical variations in the location of IC arteries and veins. For example, a tortuous vessel can be in the mid-IC space. Besides, our patient had several large-volume pleural taps on the right side signifying previous pleural trauma, which could have a propensity to bleed [1, 10].

In addition, Puchalski et al. have conducted a single-center, prospective cohort study on 312 patients with different bleeding risks. That was defined as high INR, low platelets, or using anticoagulants or anti-platelets like warfarin, heparin or clopidogrel, or having renal disease. They found that correction of bleeding risk was not necessary before thoracocentesis, as there was no significant difference in post-procedural hematocrit levels in these patients compared to the control group [4]. However, in the above studied 130 patients representing the higher bleeding-risk group, only 10 patients (8%) were on clopidogrel without other bleeding risk factors, which is a small number of patients out of the total studied. Thus, the study conclusion might not accurately apply to this subset of patients. Krysta et al. came to the same conclusion above when reviewing critical care patients’ risk of procedural bleeding [11]. The authors identified thoracocentesis as a very commonly performed procedure with a low bleeding risk (<1%), and this risk increases with obesity, complicated pleural space, and large-volume drainage, which our patient had. Clopidogrel was linked to bleeding complications in more invasive procedures such as heart rhythm device implantation. The puncture site was one of the determinants responsible for the bleeding risk in such procedures [10].

A summary of 11 published articles on the safety of using anti-platelets and anticoagulants in patients undergoing different procedures was published by Pathak et al. The authors deduced that patients might be safe to have thoracocentesis whilst on clopidogrel [1].

The latest published review article summarizing the available data on this topic concluded that it might be safe to do urgent thoracocentesis on patients using clopidogrel. However, clopidogrel is better withheld for at least five days before the procedure when possible [6].

If thoracocentesis is urgent, an experienced provider should perform the procedure under ultrasound-guidance, and after correction of other bleeding risks such as INR >2 or platelet counts <50,000 [8].

There might be some propensity towards pleural bleeding, with or without pulmonary bleeding, with clopidogrel use. A case published in the Spanish literature in 2009 reported a patient with pulmonary and pleural hemorrhage after massive clopidogrel intake as a suicidal attempt [12], which makes one wonder why bleeding occurred specifically in these body organs. Similarly, another case published in the Spanish literature in 2006 reported spontaneous, massive bilateral hemothoraces in a patient who took dual anti-platelet therapy of aspirin and clopidogrel after a coronary DES placement [13]. This also triggers further pharmacological assessment of clopidogrel sensitivity in different patients, as it is a prodrug that needs enzymatic activation in the liver by CYP2C19 [13]. Some patients lack this enzyme. Also, it is 98% protein-bound and has a significant drug-drug interaction profile. For example, some drugs inhibit the hepatic CYP2C19 enzyme thus preventing clopidogrel activation, and other drugs could potentiate its effect and add more risk of bleeding [14]. Clopidogrel’s effective half-life is approximately four hours. Thus, to rescue a bleeding patient on clopidogrel, platelets transfusion six to eight hours from the last dose can provide new functional platelets that are not inhibited by the drug [8].

## Conclusions

We support the SIR recommendations of holding clopidogrel, when feasible, five days before an elective thoracocentesis. If a patient has a recent DES insertion, then the possible risk of bleeding from the procedure should be discussed. For situations requiring emergency thoracocentesis while on clopidogrel, the patient should be counseled about possible bleeding risks as well.
